# Role of Sex Steroid Hormones in Bacterial-Host Interactions

**DOI:** 10.1155/2013/928290

**Published:** 2012-12-24

**Authors:** Elizabeth García-Gómez, Bertha González-Pedrajo, Ignacio Camacho-Arroyo

**Affiliations:** ^1^Facultad de Química, Departamento de Biología, Universidad Nacional Autónoma de México, Mexico, DF 04510, Mexico; ^2^Departamento de Genética Molecular, Instituto de Fisiología Celular, Universidad Nacional Autónoma de México, Mexico, DF 04510, Mexico

## Abstract

Sex steroid hormones play important physiological roles in reproductive and nonreproductive tissues, including immune cells. These hormones exert their functions by binding to either specific intracellular receptors that act as ligand-dependent transcription factors or membrane receptors that stimulate several signal transduction pathways. The elevated susceptibility of males to bacterial infections can be related to the usually lower immune responses presented in males as compared to females. This dimorphic sex difference is mainly due to the differential modulation of the immune system by sex steroid hormones through the control of proinflammatory and anti-inflammatory cytokines expression, as well as Toll-like receptors (TLRs) expression and antibody production. Besides, sex hormones can also affect the metabolism, growth, or virulence of pathogenic bacteria. In turn, pathogenic, microbiota, and environmental bacteria are able to metabolize and degrade steroid hormones and their related compounds. All these data suggest that sex steroid hormones play a key role in the modulation of bacterial-host interactions.

## 1. Introduction

Sex steroid hormones such as progesterone, estradiol, and testosterone play a number of important physiological roles including reproduction, differentiation, development, cell proliferation, apoptosis, inflammation, metabolism, homeostasis, and brain function [1]. They are mainly synthesized by gonads, the adrenal gland, and the placenta and are released into the blood stream to act both in peripheral target tissues and the central nervous system [2]. Sex steroid hormones exert their function by binding to either specific intracellular receptors that act as ligand-dependent transcription factors (classical mechanism) or membrane receptors that stimulate several signal transduction pathways (nonclassical mechanism) [1, 3–5].

Interestingly, sex steroid hormones also participate in the communication between microorganisms and mammal hosts. This type of communication is commonly referred to as “interkingdom signaling” and can be used by microbial pathogens to activate their virulence factors and control the course and outcome of infection [6]. Notably, human and animal males, in general, are more susceptible to protozoan, fungal, bacterial, and viral infections than females [7]. This susceptibility could be due to the lower immune responses presented in males than in females, since innate responses, antibody-mediated, and cellular responses are typically lower in males than in females [7–9].

Numerous studies have reported the effects of sex steroid hormones on the dimorphic sex differences in the response to microbial and viral infections. In addition to affecting host immunity, sex hormones alter gene expression and behavior that influence susceptibility and resistance to infection [7]. This paper mainly focuses on the participation of sex hormones in the interaction between pathogenic bacteria and their hosts, their involvement in the host mechanisms used to minimize and eradicate the infection, as well as in the pathways used by bacteria to evade the immune response.

## 2. Mechanism of Action of Sex Steroid Hormones

Many actions of estradiol, progesterone, and testosterone are mediated by the classical or genomic mechanism of action that involves specific intracellular receptors, ER, PR, and AR, respectively, which are members of the nuclear receptor superfamily of ligand-dependent transcription factors [11, 12]. Two PR isoforms have been reported in humans, which are encoded by the same gene but regulated by distinct promoters. These isoforms are PR-B of 114 kDa and an N-terminal truncated form, PR-A of 94 kDa [13]. There also exist two subtypes of ER, ER-*α* of 66 kDa and ER-*β* of 55 kDa, which are transcribed from different genes [14]. Similarly, there are two isoforms of AR encoded by a single gene, AR-A and AR-B; the latter has a molecular mass of 110 kDa, while the former has a molecular mass of 87 kDa and lacks the first 187 amino acids of the N-terminal region of AR-B [15].

**Figure 1:**
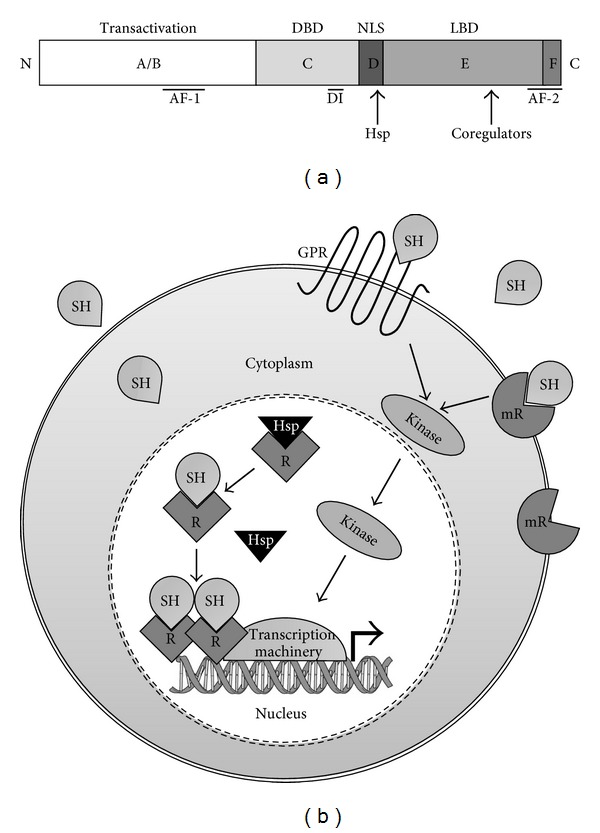
Mechanisms of action of sex steroid hormones. (a) Schematic representation of the main functional domains of sex steroid intracellular receptors. Transactivation domain (A/B) contains a transcriptional activation function (AF1). The C domain contains the DNA-binding domain (DBD) and a dimerization interface (DI). The hinge region (D domain) contains the nuclear localization signal (NLS) and binding sites for chaperones (Hsp). The ligand-binding domain (LBD) is contained in the E domain, which also contains part of AF-2 region and a site for coregulators association. The F domain includes part of AF-2. Domains are not represented to scale, modified from [10]. (b) Classical and nonclassical mechanisms of action of sex steroid hormones. Through the classical mechanism, sex hormones (SHs) exert their function by binding to specific intracellular receptors (R). In the absence of ligand, receptors are associated with heat-shock proteins (Hsps); when the hormone interacts with its specific intracellular receptor, it induces conformational changes that allow the dissociation of Hsp, promoting dimerization, phosphorylation, and receptor binding to hormone response elements located in the promoter region of target genes. Then, receptors act as ligand-dependent transcription factors, recruit coregulators, and associate to the basal transcription machinery. Alternatively, through a nonclassical mechanism, sex hormones bind to membrane receptors (mRs) that in many cases are coupled to G proteins, which stimulate several signal transduction pathways, for example, through kinase activation, modified from [11].

Sex steroid receptors are modular proteins with distinct functional domains (Figure 1(a)). The N-terminal region contains the A/B domain that has the transcriptional activation function (AF)-1. The middle region (C domain) contains the DNA-binding domain (DBD) that is the highest conserved and the dimerization region. The C domain is followed by a hinge region (D-domain) that contains a nuclear localization signal (NLS) and the binding sites for chaperone proteins that maintain receptors in an inactive state. The E domain contains the ligand-binding region (LBD), a second AF domain (AF-2) as well as a region for coregulators association. Finally, the F domain is located at the extreme C-terminal region and contains part of the AF-2 domain [10, 11, 16] (Figure 1(a)).

According to the classical model of steroid receptors action, in the absence of ligand, nuclear receptors are associated with the heat-shock proteins Hsp70 and Hsp90. When the hormone interacts with its specific intracellular receptor, it induces conformational changes that allow dissociation of Hsp70 and Hsp90 promoting dimerization, phosphorylation, and high affinity binding to hormone response elements (HREs) located in the promoter region of target genes. Then, receptors modulate transcription by recruiting components of the basal transcriptional machinery. Sex hormone receptors also mediate transcriptional activity by recruiting a group of coactivator and adapter proteins, which function as acetyl transferases, ligases, ATPases, methylases, cell cycle regulators, RNA helicases, and docking proteins to bridge to basal transcription factors. In addition to coactivators, several corepressors have been characterized that activate a family of histone deacetylases, which activity results in failure to recruit the basal transcription machinery and inhibition of gene expression [5, 11] (Figure 1(b)).

Besides the classical mechanism of action, sex steroids can act in the cells through the nonclassical or nongenomic mechanism of action, in most cases mediated by membrane receptors. Thus, membrane progesterone receptors (mPRs) have been identified. Progesterone induces rapid responses in target cells such as spermatozoids, neurons, myometrial cells and immune cells through interaction with its mPRs, and mediates signaling via G-protein-coupled pathways [17]. Estradiol can associate with the transmembrane G-protein-coupled estrogen receptor-1 (GPR30) activating the trimeric G-protein. GPR30 plays an important role in the cardiovascular and immunological systems [18, 19]. G-protein-coupled receptors for androgens have also been identified in several cell types, including breast and prostate tumor cells, vascular and immune cells [20] (Figure 1(b)). 

The signaling pathways of the nongenomic actions of sex steroids involve ion channels, enzyme-linked receptors, cyclic AMP and cyclic GMP production, mitogen-activated protein kinases (MAPKs), tyrosine kinases, and lipid kinases cascades (Figure 1(b)) [21–24]. Thus, progesterone modifies calcium influx in spermatozoa by opening membrane Ca^+2^ channels and activating the Src/p21^ras^/ERK kinase pathway. Besides, progesterone can activate MAPK pathway in different cell types [21, 25, 26]. Testosterone can depolarize Sertoli cells and cause calcium influx through inhibition of K^+^ATP channels; this hormone can also activate MAPK cascades through activation of the kinases Ras, Raf, MEK (mitogen-activated protein kinase/ERK kinase), and ERK (extracellular-signal-regulated kinase) [27]. In the case of estradiol, it can interact with GPRs in vascular cells, which activate the Src kinase that phosphorylates the epidermal growth factor receptor (EGFR) and releases metalloproteases, which trigger the release of EGF ligand from heparin. Then, EGF binds to EGFR, activating the Ras/Raf/MEK/ERK kinase system [11]. 

## 3. Modulation of Immune Responses by Sex Steroid Hormones

Sex steroid hormones markedly regulate the activity of immune cells, including lymphocytes, macrophages, granulocytes, and mast cells. The modulation of the immune system by sex steroids has both physiological and pathological implications [8, 9].

Androgen receptors have been identified in various lymphoid tissues, including the thymus and bone marrow, as well as in the spleen and in macrophages [8]. It has been reported that testosterone reduces natural killer (NK) cell activity in mice [28] and the synthesis of proinflammatory cytokines, including the tumor necrosis factor-alpha (TNF*α*) through the inhibition of transcriptional factors such as the nuclear factor kappa B (NF*κ*B) [29], whereas this hormone increases the synthesis of anti-inflammatory cytokines such as interleukin 10 (IL-10) [30]. Testosterone also decreases the expression of macrophage and monocyte Toll-like receptor 4 (TLR4), which is grouped in a family of pattern recognition receptors (PRRs) and is involved in the activation of the innate immune system in response to pathogen challenge [31]. 

On the other hand, estrogens can enhance cell-mediated and humoral immune responses. ERs are expressed in various lymphoid tissue cells as well as in circulating lymphocytes and macrophages [8]. Estradiol contributes to resistance against infections by enhancing NK cell cytotoxicity and stimulating the synthesis of proinflammatory cytokines such as IL-1, IL-6, and TNF*α* [32, 33]. Estradiol also inhibits the production of IL-4, IL-10, transforming growth factor beta (TGF-*β*) and interferon gamma (IFN-*γ*) [34, 35]. Additionally, estrogens may protect immune cells against apoptosis [36]. 

PRs have been identified in epithelial cells, mast cells, granulocytes, macrophages, and lymphocytes [8]. Progesterone is typically known as an immunosuppressive agent since it inhibits the activation of NF*κ*B and increases the expression of the suppressor of cytokine signaling protein (SOCS1) in macrophages [37]. Progesterone also reduces macrophage and NK cell activity [33, 38, 39] as well as antibody production by B cells [40]. Elevated concentrations of progesterone during pregnancy inhibit the development of Th1 (helper T-cell immune type 1) responses and the production of proinflammatory cytokines such as IFN-*γ*, while promoting Th2 immune responses, including the synthesis of anti-inflammatory cytokines such as IL-4, IL-5, and IL-10 [41].

## 4. Effects of Sex Steroid Hormones on Bacterial Infections 

Different studies provide evidence that males exhibit greater susceptibility to bacterial challenge than their female counterparts [42]. Experimental models of infection in castrated animals with or without hormonal substitution have been used to study the role of sex hormones in bacterial infections [43].

An approximation to determine the effects of sex hormones over bacterial infection has been the endotoxin lipopolysaccharide (LPS) administration to experimental animals to reproduce sepsis. Sepsis is driven by the overproduction of cytokines such as TNF-*α*, IL-1*β*, and IL-6 by macrophages, which detect bacteria and endotoxins via TLRs [44]. Circulating levels of these cytokines are higher in sepsis male patients and mice than their female equivalents, while levels of IL-10 are higher in female than in male patients or male mice treated with LPS [45, 46]. There is evidence that estradiol administration increases survival by decreasing the oxidative stress along the rat gastrointestinal tract following intraperitoneal LPS challenge [47]. In line with this observation, the removal of endogenous estrogens following ovariectomy increases mortality associated with LPS challenge in rats, and this effect was reverted by estrogens treatment. Besides, androgenized females have a higher rate of mortality following LPS administration [48]. 

Mycobacterial infections occur more frequently in males than in females. This is the case of *Mycobacterium tuberculosis* that produces a higher number of tuberculosis cases in men in all regions of the world, phenomenon that may involve sex hormones [49]. Male mice infected with* M. marinum *are more susceptible than females to mortality and bacterial colonization of lungs and spleen. When exogenous testosterone is administered, the susceptibility of female mice to infection increases, whereas castration in males attenuates the infection, demonstrating that testosterone is responsible for the increased susceptibility to *M. marinum *infection [50]. 

It has been demonstrated that estradiol and progesterone alter the gastric mucosal response to early *H. pylori* infection in ovariectomized gerbils, modifying the mucosa turnover. Progesterone-treated gerbils presented less gastritis, and a synthetic progesterone derivative (17-*α*-hydroxyprogesterone caproate) impairs the viability of *H. pylori* [51].

Another example of predisposition to infections in males is seen during Q fever, a zoonotic infection caused by *Coxiella burnetti*, which is considered a potential biological weapon. Men show symptoms, such as flu-like syndrome, pneumonia, hepatitis, myocarditis, pericarditis, meningitis, or encephalitis, more often than women. When mice were infected with *C. burnetii*, it was observed that bacterial load and granuloma number in spleen were higher in males than in females. Ovariectomized mice showed increased bacterial load in the spleen and liver, whereas the treatment of ovariectomized mice with estradiol reduced it [52].

Sex steroid hormone effects on diseases produced by bacteria depend on the infective species and sex steroid hormone levels. In contrast with the data presented above, there are bacterial infections with major incidence in women and female animal models. For example, in mice infected with *Pseudomonas aeruginosa*, indicators such as weight loss, bacterial load, and inflammatory mediators in the lung were higher in females than in males, suggesting a possible role of estrogens in female predisposition to infection by *P. aeruginosa *[53]. In support of this hypothesis, it has been observed that the administration of estradiol to male mice with pneumonia caused by *P. aeruginosa* leads to more severe inflammation in lung tissue and an increased expression of IL-17 and IL-23 [54]. 

It has been reported that female propensity to typhoid infection is due to estrogens, since the treatment with estradiol increases female mice susceptibility to an intraperitoneal *Salmonella typhimurium* challenge, whereas the treatment with progesterone increases the resistance to the infection and the survival time, suggesting a differential role of ovarian sex hormones in this infection [55]. Pregnant mice infected with *S. enterica* serovar Typhimurium showed a higher bacterial load in the spleen than nonpregnant mice, which correlates with a diminished splenic recruitment of dendritic cells, neutrophils, and NK cells, a decrease in IL-12 production, and increased levels of IL-6 [56].

Another example is the susceptibility of women to *Listeria monocytogenes *infection during pregnancy when estradiol and progesterone levels are very high [42]. Also during pregnancy, gingivitis and pyogenic granuloma have been related to the increased concentrations of circulating estrogens and progesterone [57]. As it can be observed, there is a clear sexual dimorphism in the susceptibility and progress of bacterial infections in human patients and rodent models of disease that are related with sex steroid hormone actions [42, 58]. 

Besides its role in the modulation of the immune system, sex steroid hormones have a direct effect over bacterial metabolism, growth, and expression of virulence factors. For instance, during pregnancy, the proportion of certain bacterial species associated with plaque microbiota is altered with a significant increase in the ratio of anaerobic to facultative bacteria [59]. *Prevotella intermedius* (previously *Bacteroides melaninogenicus *subsp. *intermedius *[60]) is found among these anaerobic bacteria, and interestingly, it uptakes estradiol and progesterone, which in turn enhance bacterial growth. Additionally, these sex hormones can act as substitutes for vitamin K, an essential growth factor for *P. intermedius *[59].

It has also been demonstrated that progesterone (32–127 *μ*M) inhibits the growth of *Neisseria gonorrhoeae *and *N. meningitidis*. This effect was either bacteriostatic or bactericidal, depending on progesterone concentration [61]. Interestingly, it has been shown that during infection of primary cervical epithelial cells, the treatment with progesterone (30 nM) increases *N. gonorrhoeae* survival and replication through subversion of the activity of the host serine-threonine kinase Akt by the gonococcal phospholipase D [62]. This opposite effect of progesterone could be due to the different doses of the hormone used in each study.

Studies using mouse, rat, and guinea pig models of genital tract *C. trachomatis* infection suggest that the hormonal status of the genital tract epithelium influences the outcome of the *Chlamydia trachomatis* infection [63]. In an *in vitro* model of infection of HeLa cells with *C. trachomatis*, estradiol preexposition of cells enhances the adherence of chlamydial elementary bodies, as well as the development of bacterial inclusions [64]. Recently, it was demonstrated that the persistence phenotype, defined as a long-term association between *Chlamydia* and their host cell in which the bacteria remain viable but nonculturable, also occurs in response to high levels of sex hormones, in particular estradiol that regulates the expression of genes related to persistence. For example, estradiol exposure results in the upregulation of the *trpB* gene, a marker for chlamydial persistence. Progesterone administration resulted in a general upregulation of genes that encode elements of carbohydrate and amino acid metabolism pathways [63]. These observations constitute an evidence of the direct influence of sex steroid hormones over expression of factors involved in virulence of a bacterial pathogen and particularly in the development of persistence. 

Recently, a strain of *P. aeruginosa* isolated from the lung of a woman with cystic fibrosis showed an increased production of alginate (an extracellular polymer involved in biofilm development) in the presence of estradiol, which correlates with the exacerbation of cystic fibrosis occurring at the end of the follicular phase when levels of estradiol are high [65]. 

**Figure 2:**
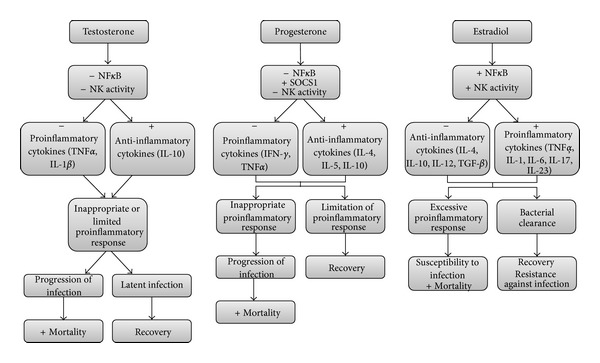
Effects of sex steroid hormones on bacterial infections. In general, male mammals are more susceptible to bacterial infections and its negative outcomes than their female counterparts. This is due to the suppressor effect of testosterone on the immune system, while estradiol acts as an activator of the immune system. Testosterone reduces the NK cell activity and induces the production of anti-inflammatory cytokines such as IL-10, whereas it reduces the production of proinflammatory cytokines such as TNF*α* through the inhibition of NF*κ*B. This conduces to an inappropriate proinflammatory response that in turn allows the progression of the infection and its negative effects, such as an increase in mortality. In some cases, the limited proinflammatory response leads to a latent infection that can be abated and conduces to recovery. Progesterone acts as a modulator of the immune system due to its suppressing effects by reducing the NK cell activity, inducing the production of IL-4, IL-5 and IL-10 and increasing the expression of SOCS1, while inhibiting the production of IFN*γ* and TNF*α*, which avoid the development of bacterial infections, subsequent bacteremia, and sepsis. However, in high levels, for example during pregnancy, progesterone predisposes to some bacterial infections due to reduced proinflammatory responses. On the other hand, estradiol enhances the NK cell activity, and through the activation of NF*κ*B, induces the production TNF*α*, IL-1, IL-6. IL-17, and IL-23, while inhibiting the production of IL-4, IL-10, IL-12, and TGF-*β*, and allows the bacterial clearance and recovery from infection. However, estradiol can also produce an excessive proinflammatory response and increased mortality as a consequence of susceptibility to infection and multiple organ failure. +, increase; −, reduction.

Germination rate of spores of *Clostridium sordellii*, a bacterium that produces hemorrhagic enteritis in several animals as well as infections of the human female genital tract during postpregnancy, is increased in response to progesterone. In contrast, it acts as an inhibitor of germination of spores of *C. difficile*, which is a gut pathogen associated with diarrhea. In this case, progesterone competes with bile salt taurocholate that is recognized as a germinant, probably by binding to the same receptors that recognize taurocholate in *C. difficile* spores. This is an example of how spores of two related species differentially respond to sex steroids [66]. The effects of sex steroid hormones on bacterial infections are summarized in Figure 2.

## 5. Bacterial Metabolism of Sex Steroid Hormones

Bacteria are capable of metabolizing sex steroid hormones through the activity of distinct enzymes such as hydroxysteroid dehydrogenase (HSD) that regulate the balance between active and inactive steroids. Bioinformatics analyses have identified genes that encode HSDs in distinct bacterial genomes. The dominating phyla that were identified to express these enzymes were Actinobacteria, Proteobacteria, and Firmicutes. A large number of HSD-expressing bacteria constitute the normal gastrointestinal microbiota, while another group of bacteria were isolated from natural habitats like seawater, soil, and marine sediments [67]. 

In regard to pathogenic bacteria, *Prevotella intermedius *(previously *Bacteroides melaninogenicus* subsp. *intermedius*), a gingival infective agent, uptakes progesterone and estradiol [59], while *Streptococcus mutans and Bacillus cereus* metabolize testosterone and progesterone due to the activity of 5*α*-steroid reductase 3*β*-, 17*β*-, and 20*α*-HSDs and steroid hydroxylases produced by* B. cereus*, whereas *S. mutans* produces 5*α*- and 5*β*-steroid reductases and 3*α*-, 17*β*-, and 20*α*-HSDs [68]. *Porphyromonas gingivalis* and *Actinobacillus actinomycetemcomitans* also reduce testosterone to 5*α*-dihydrotestosterone [69]. 

*Treponema denticola,* another gingival bacterium associated with periodontitis, metabolizes cholesterol, progesterone, and testosterone using 5*α*-reductase, 3*β*- and 17*β*-HSD activity [70]. However, only cholesterol induces bacterial growth, whereas high concentrations of progesterone and testosterone inhibit it. The lack of sensitivity of *T. denticola* to low concentrations of progesterone and testosterone (0.0001 *μ*g/mL) may be due to their active removal by an ATP-binding cassette (ABC) efflux transporter [71].

It has been reported that sex steroid hormones are substrates of *E. coli* multidrug efflux (MDE) pumps that are important factors in the resistance against bile acids. Two of these MDE systems, AcrAB-TolC and EmrAB-TolC, can transport estradiol and progesterone outside the bacterial cell. Additionally, when both systems were mutated, a steroid hormone-dependent growth suppression was observed [72]. Likewise, in *N. gonorrhoeae, *it has been demonstrated the participation of an efflux pump (MtrCDE) in the transport of sex hormones, which confers bacterial resistance to progesterone [73]. Efflux-deficient gonococcal mutants were more rapidly cleared from infected intact female mice than from ovariectomized mice and were more sensitive to progesterone *in vitro* [73]. These pumps may be essential for bacterial survival under conditions where steroids are present, such as in the gastrointestinal, vaginal, and urinary tracts [72].

Pathogenic bacteria also have an influence over host sex hormone metabolism. For instance, *S. enterica* infection in a murine model reduces the levels of steroid hormones such as progesterone. The analysis of the transcript levels of genes that encode several enzymes involved in the synthesis of steroid hormones reveals that the expression of some HSDs is reduced [74].

In addition to bacterial pathogens, bacteria from human microbiota play an important role in the metabolism of sex hormones. Microbiota is critical for human health since it has been implicated in the development of immune system, energy homeostasis, and protection against pathogens. Moreover, imbalances in the intestinal microbiota have also been associated with pathological processes [75]. A known cause of intestinal microbiota alteration is the use of antibiotics that can increase the susceptibility to enteric infections [76]. In a recent metabolomics study, it has been determined that the treatment of mice with streptomycin disrupts the intestinal homeostasis, through a reduction in the number of fecal bacteria and consequently by affecting the intestinal metaboloma. 87% of all metabolites detected were diminished, including steroids, suggesting that the intestinal microbiota is involved in steroid metabolism [75].

It has been observed that fecal bacteria can perform hydrolytic, reductive, and oxidative reactions of androgens and estrogens [77]. Enzymes involved in 21-dehydroxylation or 16*α*-dehydroxylation of steroids such as corticosteroids and sex hormones have been identified in intestinal microbiota, and interestingly, these enzymes are not present in mammalian tissues [78]. Reversible 17*β* reduction of androgens carried out by human intestinal microorganisms is suggested to play a role in the regulation of testosterone levels and in the release of androgens in humans [78, 79].

Sex steroid metabolism is not only carried out by pathogenic or microbiota bacteria, but also by environmental bacteria, such as soil-, marine-, and sludge-associated organisms. The most studied example of steroid metabolism and steroid-dependent gene regulation in bacteria is the soil bacterium *Comamonas testosteroni* (formerly *Pseudomonas testosteroni*) [80]. *C. testosteroni* expresses various genes that respond to steroids including receptors such as TeiR, as well as activators (TesR) and repressors (RepA and RepB) of the 3*α*-HSD/carbonil reductase (CR-) encoding gene, *hsdA*. These proteins participate in the adaptation of the bacteria to the environment [81, 82]. Particularly, 3*α*-HSD/CR is an enzyme involved in the metabolism of androgens that mediates the oxide reduction of androstenedione, 5*α*-dihydrotestosterone, and androsterone. Interestingly, the expression of 3*α*-HSD/CR is highly inducible in the presence of steroid substrates [67, 83].

A testosterone catabolic pathway that differs to that found in *C. testosteroni *has been described in *Steroidobacter denitrificans *[84], a bacterium isolated from sludge that is capable of metabolizing estradiol and testosterone [85]. This bacterium oxidizes testosterone to 1-dehydrotestosterone, which is then transformed into androsta-1,4-diene-3,17-dione that in turn undergoes a reduction reaction occurring at its A ring; probably this reaction is accomplished by an as yet unidentified 3*α*-HSD [84]. Some seawater bacteria can also degrade steroids, for instance the marine bacterium H5, belonging to the genus *Vibrio*, can degrade testosterone and estrogens. Additionally, two estradiol inducible genes coding a 3-ketosteroid-Δ-1-dehydrogenase and a carboxylesterase were identified [86]. 

Since natural and synthetic steroid hormones released into the environment are a potential health risk to humans and animals by interfering with sexual development and reproduction, among other functions, steroid-degrading bacterial species may be useful in the bioremediation of contaminated environments, process also known as bioaugmentation [67]. The latter has been successfully applied in a variety of environments and in degradation of different pollutants such as petroleum hydrocarbon, phenol, and the herbicide atrazine [87].

Estradiol and its primary degradation product estrone have been detected in surface water, groundwater, livestock, and municipal wastes. The majority of bacteria that degrade estradiol such as *Bacillus amyloliquefaciens, B. subtilis,* and *B. cereus* have been isolated from sludge and can convert estradiol into estrone, but they cannot further degrade estrone [88]. Other estradiol-degrading bacteria isolated from activated sludge of a wastewater treatment plant that can be used in bioremediation of polluted environments correspond to genera *Aminobacter*, *Brevundimonas*, *Escherichia*, *Flavobacterium*, *Microbacterium*, *Nocardioides*, *Rhodococcus,* and *Sphingomonas. *Most of these strains cannot further degrade estrone [89]. 

In *Stenotrophomonas maltophilia*, a bacterium that degrades estradiol, it was determined that estrone is converted into tyrosine through the cleavage of its saturated ring, this amino acid in turn can be utilized in protein biosynthesis; however, the enzyme responsible of this conversion was not identified [90]. *Sphingomonas *strain KC8, whose genome sequence has been recently reported [91], has the capability of degrading different steroids, such as estradiol, estrone, and testosterone [92]. Although the degradation mechanism used by this bacterium has not been identified, its genome contains several genes encoding the enzymes putatively involved in estrogen degradation, such as HSD, 3-ketosteroid-Δ-1-dehydrogenase and catechol 2,3-dioxygenase [91]. Another bacterium of the *Sphingomonadaceae* family, named EDB-LI1, forms biofilms and it also degrades estrone [87].

The identification of key enzymes in biodegradation could help to discover microbial estrogen degradation pathways and suggest biomarkers to monitor estrogen degradation by a microbial community [90], which can be constituted by a mixture of distinct bacteria capable of degrading various classes of steroid hormones and their related compounds.

## 6. Conclusions

Sex steroid hormones play important roles in diverse functions of mammals, such as the modulation of the immune response. Testosterone, estradiol, and progesterone can differentially regulate responses against bacterial infections and alter metabolic pathways of pathogenic and microbiota bacteria. In general, testosterone acts as an immunosupressor, while estradiol acts as an activator, and progesterone acts as a modulator of the immune system. These effects are related to the sexual dimorphism found in bacterial infections, where men and male animals are in many cases more susceptible to bacterial infections than females. The stage of the menstrual or estrous cycles and pregnancy also determines the outcome of bacterial infections due to the changes in the levels of sex hormones. In some cases, administration of sex hormones may control the course of bacterial infections, functioning as a complement to antibiotic therapy. In turn, bacteria have developed mechanisms to eliminate or to exploit sex hormones in their benefit by using them as carbon and energy sources, principally through their degradation or chemical modification. Interestingly, this feature can be utilized in human benefit by using bacteria capable of degrading and eliminating steroid hormones from polluted environments. The knowledge of the specific enzymes and mechanisms involved in these processes could be helpful in the selection of appropriate bacteria to be used in bioremediation programs.

## References

[1] Edwards DP (2005). Regulation of signal transduction pathways by estrogen and progesterone. *Annual Review of Physiology*.

[2] Wilson JW, Foster DW, Kronenberg H, Larsen PR, Wilson JW (1998). Principles of endocrinology. *William Textbook of Endocrinology*.

[3] Scarpin KM, Graham JD, Mote PA, Clarke CL (2009). Progesterone action in human tissues: regulation by progesterone receptor (PR) isoform expression, nuclear positioning and coregulator expression. *Nuclear Receptor Signaling*.

[4] Patrão MT, Silva EJ, Avellar MC (2009). Androgens and the male reproductive tract: an overview of classical roles and current perspectives. *Arquivos Drasileiros de Endocrinologia e Metabologia*.

[5] Cabrera-Muñoz E, Hernández-Hernández OT, Camacho-Arroyo I (2012). Role of estradiol and progesterone in HIV susceptibility and disease progression. *Mini-Reviews in Medicinal Chemistry*.

[6] Hughes DT, Sperandio V (2008). Inter-kingdom signalling: communication between bacteria and their hosts. *Nature Reviews Microbiology*.

[7] Klein SL (2000). The effects of hormones on sex differences in infection: from genes to behavior. *Neuroscience and Biobehavioral Reviews*.

[8] Klein SL (2004). Hormonal and immunological mechanisms mediating sex differences in parasite infection. *Parasite Immunology*.

[9] Ahmed SA, Karpuzoglu E, Khan D, Klein SL, Roberts CW (2010). Effects of sex steroids on innate and adaptive immunity. *Sex Hormones and Immunnity To Infection*.

[10] Camacho-Arroyo I, Rodríguez-Dorantes M, Joseph-Bravo P (2006). Transcriptional activity regulated by progesterone receptor isoforms. *Molecular Endocrinology*.

[11] Wierman ME (2007). Sex steroid effects at target tissues: mechanisms of action. *American Journal of Physiology—Advances in Physiology Education*.

[12] Cabrera-Muñoz A, Escobedo G, Guzmán C, Camacho-Arroyo I (2010). Role of progesterone in HIV and parasitic infections. *The Open Neuroendocrinology Journal*.

[13] Kastner P, Krust A, Turcotte B (1990). Two distinct estrogen-regulated promoters generate transcripts encoding the two functionally different human progesterone receptor forms A and B. *The EMBO Journal*.

[14] MacGregor JI, Jordan VC (1998). Basic guide to the mechanisms of antiestrogen action. *Pharmacological Reviews*.

[15] Wilson CM, McPhaul MJ (1994). A and B forms of the androgen receptor are present in human genital skin fibroblasts. *Proceedings of the National Academy of Sciences of the United States of America*.

[16] Bain DL, Heneghan AF, Connaghan-Jones KD, Miura MT (2007). Nuclear receptor structure: implications for function. *Annual Review of Physiology*.

[17] Zhu Y, Hanna RN, Schaaf MJM, Spaink HP, Thomas P (2008). Candidates for membrane progestin receptors-Past approaches and future challenges. *Comparative Biochemistry and Physiology C*.

[18] Thomas P, Pang Y, Filardo EJ, Dong J (2005). Identity of an estrogen membrane receptor coupled to a G protein in human breast cancer cells. *Endocrinology*.

[19] Mizukami Y (2010). In vivo functions of GPR30/GPER-1, a membrane receptor for estrogen: from discovery to functions in vivo. *Endocrine Journal*.

[20] Papadopoulou N, Papakonstanti EA, Kallergi G, Alevizopoulos K, Stournaras C (2009). Membrane androgen receptor activation in prostate and breast tumor cells: molecular signaling and clinical impact. *IUBMB Life*.

[21] Simoncini T, Genazzani AR (2003). Non-genomic actions of sex steroid hormones. *European Journal of Endocrinology*.

[22] Castillo C, Ceballos G, Rodríguez D (2006). Effects of estradiol on phenylephrine contractility associated with intracellular calcium release in rat aorta. *American Journal of Physiology—Cell Physiology*.

[23] Benten WPM, Guo Z, Krücken J, Wunderlich F (2004). Rapid effects of androgens in macrophages. *Steroids*.

[24] Carreau S, Bouraima-Lelong H, Delalande C (2011). Estrogens: new players in spermatogenesis. *Reproductive Biology*.

[25] Mani SK, Oyola MG (2012). Progesterone signaling mechanisms in brain and behavior. *Frontiers in Endocrinology*.

[26] González-Flores O, Shu J, Camacho-Arroyo I, Etgen AM (2004). Regulation of lordosis by cyclic 3′,5′-guanosine monophosphate, progesterone, and its 5*α*-reduced metabolites involves mitogen-activated protein kinase. *Endocrinology*.

[27] Walker WH (2010). Non-classical actions of testosterone and spermatogenesis. *Philosophical Transactions of the Royal Society B*.

[28] Hou J, Zheng WF (1988). Effect of sex hormones on NK and ADCC activity of mice. *International Journal of Immunopharmacology*.

[29] McKay LI, Cidlowski JA (1999). Molecular control of immune/inflammatory responses: interactions between nuclear factor-*κ*B and steroid receptor-signaling pathways. *Endocrine Reviews*.

[30] Agostino PD', Milano S, Barbera C (1999). Sex hormones modulate inflammatory mediators produced by macrophages. *Annals of the New York Academy of Sciences*.

[31] Rettew JA, Huet-Hudson YM, Marriott I (2008). Testosterone reduces macrophage expression in the mouse of toll-like receptor 4, a trigger for inflammation and innate immunity. *Biology of Reproduction*.

[32] Sorachi KI, Kumagai S, Sugita M, Yodoi J, Imura H (1993). Enhancing effect of 17*β*-estradiol on human NK cell activity. *Immunology Letters*.

[33] Miller L, Hunt JS (1996). Sex steroid hormones and macrophage function. *Life Sciences*.

[34] Straub RH (2007). The complex role of estrogens in inflammation. *Endocrine Reviews*.

[35] Salem ML, Hossain MS, Nomoto K (2000). Mediation of the immunomodulatory effect of *β*-estradiol on inflammatory responses by inhibition of recruitment and activation of inflammatory cells and their gene expression of TNF-*α* and IFN-*γ*. *International Archives of Allergy and Immunology*.

[36] Vegeto E, Pollio G, Pellicciari C, Maggi A (1999). Estrogen and progesterone induction of survival of monoblastoid cells undergoing TNF-*α*-induced apoptosis. *FASEB Journal*.

[37] Su L, Sun Y, Ma F, Lü P, Huang H, Zhou J (2009). Progesterone inhibits Toll-like receptor 4-mediated innate immune response in macrophages by suppressing NF-*κ*B activation and enhancing SOCS1 expression. *Immunology Letters*.

[38] Savita, Rai U (1998). Sex steroid hormones modulate the activation of murine peritoneal macrophages: receptor mediated modulation. *Comparative Biochemistry and Physiology C*.

[39] Furukawa K, Itoh K, Okamura K (1984). Changes in NK cell activity during the estrous cycle and pregnancy in mice. *Journal of Reproductive Immunology*.

[40] Lü FX, Abel K, Ma Z (2002). The strength of B cell immunity in female rhesus macaques is controlled by CD^8+^ T cells under the influence of ovarian steroid hormones. *Clinical and Experimental Immunology*.

[41] Robinson DP, Klein SL (2012). Pregnancy and pregnancy-associated hormones alter immune responses and disease pathogenesis. *Hormones and Behavior*.

[42] Rettew JA, Marriot I, Huett YM, Klein SL, Roberts CW (2010). Sex differences in innate immune responses to bacterial pathogens. *Sex Hormones and Immunity To Infection*.

[43] Leone M, Textoris J, L CCapoMJ, Dubey RK (2012). Sex hormones and bacterial infections. *Sex Hormones*.

[44] Blackwell TS, Christman JW (1996). Sepsis and cytokines: current status. *British Journal of Anaesthesia*.

[45] Marriott I, Bost KL, Huet-Hudson YM (2006). Sexual dimorphism in expression of receptors for bacterial lipopolysaccharides in murine macrophages: a possible mechanism for gender-based differences in endotoxic shock susceptibility. *Journal of Reproductive Immunology*.

[46] Schröder J, Kahlke V, Staubach KH, Zabel P, Stüber F (1998). Gender differences in human sepsis. *Archives of Surgery*.

[47] Şener G, Arbak S, Kurtaran P, Gedik N, Yeğen BÇ (2005). Estrogen protects the liver and intestines against sepsis-induced injury in rats. *Journal of Surgical Research*.

[48] Merkel SM, Alexander S, Zufall E, Oliver JD, Huet-Hudson YM (2001). Essential role for estrogen in protection against vibrio vulnificus-induced endotoxic shock. *Infection and Immunity*.

[49] Neyrolles O, Quintana-Murci L (2009). Sexual inequality in tuberculosis. *PLoS Medicine*.

[50] Yamamoto Y, Saito H, Setogawa T, Tomioka H (1991). Sex differences in host resistance to Mycobacterium marinum infection in mice. *Infection and Immunity*.

[51] Hosoda K, Shimomura H, Hayashi S, Yokota K, Hirai Y (2011). Steroid hormones as bactericidal agents to *Helicobacter pylori*. *FEMS Microbiology Letters*.

[52] Leone M, Honstettre A, Lepidi H (2004). Effect of sex on coxiella burnetii infection: protective role of 17*β*-estradiol. *Journal of Infectious Diseases*.

[53] Guilbault C, Stotland P, Lachance C (2002). Influence of gender and interleukin-10 deficiency on the inflammatory response during lung infection with *Pseudomonas aeruginosa* in mice. *Immunology*.

[54] Wang Y, Cela E, Gagnon S, Sweezey NB (2010). Estrogen aggravates inflammation in *Pseudomonas aeruginosa* pneumonia in cystic fibrosis mice. *Respiratory Research*.

[55] Kita E, Yagyu Y, Nishikawa F (1989). Alterations of host resistance to mouse typhoid infection by sex hormones. *Journal of Leukocyte Biology*.

[56] Pejcic-Karapetrovic B, Gurnani K, Russell MS, Finlay BB, Sad S, Krishnan L (2007). Pregnancy impairs the innate immune resistance to Salmonella typhimurium leading to rapid fatal infection. *Journal of Immunology*.

[57] Ovadia R, Zirdok R, Diaz-Romero RM (2007). Relationship between pregnancy and periodontal disease. *Facta Universitatis Series Medicine and Biology*.

[58] Fish EN (2008). The X-files in immunity: sex-based differences predispose immune responses. *Nature Reviews Immunology*.

[59] Kornman KS, Loesche WJ (1982). Effects of estradiol and progesterone on *Bacteroides melaninogenicus* and *Bacteroides gingivalis*. *Infection and Immunity*.

[60] Shah HN, Collins DM (1990). Prevotella, a new genus to include *Bacteroides melaninogenicus* and related species formerly classified in the genus *Bacteroides*. *International Journal of Systematic Bacteriology*.

[61] Morse SA, Fitzgerald TJ (1974). Effect of progesterone on *Neisseria gonorrhoeae*. *Infection and Immunity*.

[62] Edwards JL (2010). *Neisseria gonorrhoeae* survival during primary human cervical epithelial cell infection requires nitric oxide and is augmented by progesterone. *Infection and Immunity*.

[63] Amirshahi A, Wan C, Beagley K, Latter J, Symonds I, Timms P (2011). Modulation of the *Chlamydia trachomatis* in vitro transcriptome response by the sex hormones estradiol and progesterone. *BMC Microbiology*.

[64] Bose SK, Goswami PC (1986). Enhancement of adherence and growth of *Chlamydia trachomatis* by estrogen treatment of HeLa cells. *Infection and Immunity*.

[65] Chotirmall SH, Smith SG, Gunaratnam C (2012). Effect of estrogen on pseudomonas mucoidy and exacerbations in cystic fibrosis. *The New England Journal of Medicine*.

[66] Liggins M, Ramirez N, Magnuson N, Abel-Santos E (2011). Progesterone analogs influence germination of *Clostridium sordellii* and *Clostridium difficile* spores in vitro. *Journal of Bacteriology*.

[67] Kisiela M, Skarka A, Ebert B, Maser E (2012). Hydroxysteroid dehydrogenases (HSDs) in bacteria: a bioinformatic perspective. *The Journal of Steroid Biochemistry and Molecular Biology*.

[68] Ojanotko-Harri A, Nikkari T, Harri MP, Paunio KU (1990). Metabolism of progesterone and testosterone by *Bacillus* cereus strain Socransky 67 and *Streptococcus mutans* strain Ingbritt. *Oral Microbiology and Immunology*.

[69] Soory M (1995). Bacterial steroidogenesis by periodontal pathogens and the effect of bacterial enzymes on steroid conversions by human gingival fibroblasts in culture. *Journal of Periodontal Research*.

[70] Clark DT, Soory M (2006). The metabolism of cholesterol and certain hormonal steroids by *Treponema denticola*. *Steroids*.

[71] Clark DT, Soory M (2006). The influence of cholesterol, progesterone, 4-androstenedione and testosterone on the growth of *Treponema denticola* ATCC 33520 in batch cultures. *Anaerobe*.

[72] Elkins CA, Mullis LB (2006). Mammalian steroid hormones are substrates for the major RND- and MFS-type tripartite multidrug efflux pumps of *Escherichia coli*. *Journal of Bacteriology*.

[73] Jerse AE, Sharma ND, Simms AN, Crow ET, Snyder LA, Shafer WM (2003). A gonococcal efflux pump system enhances bacterial survival in a female mouse model of genital tract infection. *Infection and Immunity*.

[74] Antunes LCM, Arena ET, Menendez A (2011). Impact of *Salmonella* infection on host hormone metabolism revealed by metabolomics. *Infection and Immunity*.

[75] Antunes LCM, Han J, Ferreira RBR, Lolić P, Borchers CH, Finlay BB (2011). Effect of antibiotic treatment on the intestinal metabolome. *Antimicrobial Agents and Chemotherapy*.

[76] Sekirov I, Tam NM, Jogova M (2008). Antibiotic-induced perturbations of the intestinal microbiota alter host susceptibility to enteric infection. *Infection and Immunity*.

[77] Lombardi P, Goldin B, Boutin E, Gorbach SL (1978). Metabolism of androgens and estrogens by human fecal microorganisms. *Journal of Steroid Biochemistry*.

[78] Bokkenheuser VD (1980). Biotransformation of steroid hormones by gut bacteria. *American Journal of Clinical Nutrition*.

[79] Donova MV, Egorova OV, Nikolayeva VM (2005). Steroid 17*β*-reduction by microorganisms—a review. *Process Biochemistry*.

[80] Göhler A, Xiong G, Paulsen S, Trentmann G, Maser E (2008). Testosterone-inducible regulator is a kinase that drives steroid sensing and metabolism in *Comamonas testosteroni*. *Journal of Biological Chemistry*.

[81] Watanabe M, Phillips K, Chen T (1973). Steroid receptor in *Pseudomonas testosteroni* released by osmotic shock. *Journal of Steroid Biochemistry*.

[82] Xiong G, Maser E (2001). Regulation of the steroid-inducible 3*α*-hydroxysteroid dehydrogenase/carbonyl reductase gene in *Comamonas testosteroni*. *Journal of Biological Chemistry*.

[83] Maser E, Xiong G, Grimm C, Ficner R, Reuter K (2001). 3*α*-hydroxysteroid dehydrogenase/carbonyl reductase from *Comamonas testosteroni*: biological significance, three-dimensional structure and gene regulation. *Chemico-Biological Interactions*.

[84] Leu YL, Wang PH, Shiao MS, Ismail W, Chiang YR (2011). A novel testosterone catabolic pathway in bacteria. *Journal of Bacteriology*.

[85] Fahrbach M, Kuever J, Remesch M (2008). Steroidobacter *denitrificans* gen. nov., sp. nov., a steroidal hormone-degrading gammaproteobacterium. *International Journal of Systematic and Evolutionary Microbiology*.

[86] Sang Y, Xiong G, Maser E (2011). Steroid degradation and two steroid-inducible enzymes in the marine bacterium H5. *Chemico-Biological Interactions*.

[87] Iasur-Kruh L, Hadar Y, Minz D (2011). Isolation and bioaugmentation of an estradiol-degrading bacterium and its integration into a mature biofilm. *Applied and Environmental Microbiology*.

[88] Jiang L, Yang J, Chen J (2010). Isolation and characteristics of 17*β*-estradiol-degrading *Bacillus* spp. strains from activated sludge. *Biodegradation*.

[89] Yu CP, Roh H, Chu KH (2007). 17*β*-estradiol-degrading bacteria isolated from activated sludge. *Environmental Science and Technology*.

[90] Li Z, Nandakumar R, Madayiputhiya N, Li X (2012). Proteomic analysis of 17beta-estradiol degradation by *Stenotrophomonas maltophilia*. *Environmental Science & Technology*.

[91] Hu A, He J, Chu KH, Yu CP (2011). Genome sequence of the 17*β*-estradiol-utilizing bacterium *sphingomonas* strain. *Journal of Bacteriology*.

[92] Roh H, Chu KH (2010). A 17*β*-estradiol-utilizing bacterium, *sphingomonas* strain KC8: part I—characterization and abundance in wastewater treatment plants. *Environmental Science and Technology*.

